# Perioperative airway strategy for a singer using a supraglottic airway device and bronchial blocker to prevent hoarseness following robotic lobectomy

**DOI:** 10.1186/s40981-025-00817-5

**Published:** 2025-09-26

**Authors:** Keisuke Yoshida, Kazuho Saito, Yuzo Iseki, Yoshie Noji, Satoki Inoue

**Affiliations:** Department of Anesthesiology, Fukushima Medical University School of Medicine, 1 Hikarigaoka, Fukushima City, Fukushima, 960-1295 Japan

*To the editor,*

In thoracic surgeries requiring one-lung ventilation (OLV), double-lumen tubes (DLTs) or bronchial blockers (BBs) are commonly used to achieve lung isolation. While DLTs ensure reliable isolation, their large diameter and rigidity can irritate the vocal cords, often resulting in postoperative hoarseness. The incidence of hoarseness within the first 24 h after surgery has been reported at 10–50% [1–3]. Even when a single-lumen tube and a BB are used, postoperative hoarseness has been observed in up to 17% of reported cases [3]. Here, we report a case in which use of a supraglottic airway device (SGA) combined with a BB successfully prevented postoperative hoarseness in a patient for whom voice preservation was of particular importance.

The patient was a woman in her 60s (158 cm, 54 kg) scheduled to undergo robot-assisted right lower lobectomy for lung cancer. Given that she was a singer who was in the midst of preparing for concert where she would sing alone within one month postoperatively, we opted for an airway management strategy that aimed to minimize laryngeal trauma. Instead of the standard 35Fr DLT typically used for female patients, we used a size 4 i-gel® (Intersurgical Ltd., Wokingham, UK) and a Coopdech Endobronchial Blocker (Daiken Medical Co., Ltd., Osaka, Japan), based on the method described by Nakanishi et al. [4].

The BB was pre-positioned through the ventilation port of the i-gel®. Using a McGRATH™ MAC video laryngoscope with a size 3 blade (Medtronic, Minneapolis, MN, USA), the BB was first inserted into the trachea. The i-gel® was then inserted blindly in the usual manner. Finally, the position of the BB was adjusted under bronchoscopic guidance. OLV was initiated in the left lateral decubitus position under pressure-controlled ventilation (positive end-expiratory pressure 4 cmH₂O, driving pressure 15 cmH₂O, respiratory rate 15/min, and inspiratory time 1.5 s). Adequate lung isolation was confirmed with a tidal volume of approximately 340 mL and no major air leak. The surgery proceeded uneventfully with stable OLV maintained throughout. Immediately after removal, the patient exhibited no subjective or objective signs of hoarseness or throat discomfort.

Although BBs are less efficient than DLTs in achieving lung deflation due to their narrow lumens, in robot-assisted thoracic surgery, CO₂ insufflation increases intrathoracic pressure, thereby facilitating lung collapse and compensating for this limitation. While BBs also pass through the vocal cords, their curved shape often allows them to rest in the interarytenoid space, reducing direct pressure. In addition, their slight mobility may contribute to the distribution of pressure. In the present case, we confirmed that the BB did not cause visible deformation or compression of the vocal cords. Furthermore, the use of SGAs can support smoother emergence from anesthesia, and minimize the risk of trauma to the respiratory system [5]. Regarding the selection of an SGA, we chose the i-gel because we had substantially more clinical experience with this device, and we consider that selecting the SGA most familiar to the operator is of primary importance. Although the SGA-BB combination is not yet standard practice, it may be especially advantageous in cases where voice preservation is of particular importance. We have reported this case to promote broader awareness of this technique.

## Data Availability

Not applicable.
